# Unfavorable metabolic changes are accompanied by impaired myocardial function shortly after chemotherapy

**DOI:** 10.1186/1532-429X-15-S1-O46

**Published:** 2013-01-30

**Authors:** RW Van Der Meer, Peter-Paul M Willemse, Linda Van Schinkel, Susanne Osanto, Jacobus Burggraaf, Saskia Van Elderen, Albert de Roos, Hildo J Lamb

**Affiliations:** 1 Radiology, Leiden University Medical Center, Leiden, the Netherlands; 2 Endocrinology, Leiden University Medical Center, Leiden, the Netherlands; 3 Clinical Oncology, Leiden University Medical Center, Leiden, the Netherlands; 4 Centre for Human Drug Research, Leiden, the Netherlands

## Background

Cardiovascular morbidity is a well known late complication of chemotherapy, for example for treatment of testicular cancer. In addition, development of a combination of overweight, hypertension and abnormal lipid profiles has been observed in these patients suggesting increased risk of developing the metabolic syndrome and diabetes. The early effects of chemotherapy on myocardial function and metabolic profile are largely unknown. Therefore, the purpose of this study was to assess short-term effects of chemotherapy in testicular cancer on myocardial function in relationship with alterations in metabolic profile.

## Methods

Fourteen patients with testicular cancer were treated with bleomycin, etoposide and cisplatin.

Before and after chemotherapy, magnetic resonance imaging techniques were used to assess cardiac systolic and diastolic function, and abdominal fat volume (summation of 3 slices at the level of the L5 vertebra). In addition, hepatic and myocardial triglyceride content were assessed using MR spectroscopy. Blood samples were taken at both occasions to obtain plasma lipid profile and to estimate insulin sensitivity.

## Results

After chemotherapy, an unfavorable shift in metabolic profile was observed: Visceral abdominal fat volume was increased significantly (from186 ± 125 ml to 227 ± 162 ml. P< 0.05) without significant changes in BMI. Hepatic triglyceride content increased, although non-significant (from 3.23 ± 2.72 % to 4.65 ± 4.85% P> 0.05). In addition proxy-measures of insulin sensitivity (Quicki) decreased from 0.39 ± 0.05 to 0.36 ± 0.05 P < 0.05 and serum LDL-cholesterol increased significantly after chemotherapy treatment from 3.12 ± 1.15 mmol/l to 3.74 ± 1.41 mmol/l.

These metabolic derangements were paralleled by subclinical changes in myocardial function. Diastolic function, as assessed as the E/A ratio, decreased (from 1.87 ± 0.43 to ± 1.64 ± 0.45 P< 0.05.) Furthermore, left ventricular end-diastolic volume was decreased (from192 ± 27 ml. to 175 ± 26 ml. P< 0.05), indicating disturbed ventricular relaxation. Myocardial systolic function and myocardial triglyceride content were unaltered.

## Conclusions

Chemotherapy for testicular cancer induces unfavorable metabolic changes, which are paralleled by impairment in diastolic heart function.

## Funding

None.

